# Biological Restoration of Conventional Aqueous Outflow Pathways in Glaucoma

**DOI:** 10.3390/biomedicines14102355

**Published:** 2026-10-16

**Authors:** Matteo Capobianco, Marco Zeppieri, Alessandro Avitabile, Ludovica Cannizaro, Alessandra Pizzo, Irene Gattazzo, Fabiana D’Esposito, Francesco Cappellani, Caterina Gagliano

**Affiliations:** 1 Eye Clinic, Catania University, Policlinico G. Rodolico, 95121 Catania, Italy; capobiancoteo@gmail.com; 2 Department of Ophthalmology, University Hospital of Udine, 33100 Udine, Italy; 3 Department of Medicine, Surgery and Health Sciences, University of Trieste, 34129 Trieste, Italy; 4 Biomedicine, Neuroscience and Advance Diagnostic (BIND) Department, University of Palermo, 90128 Palermo, Italy; 5 Unità Operativa Complessa di Oftalmologia, Azienda Ospedaliera per l’Emergenza “Cannizzaro”, 95026 Catania, Italy; 6 Department of Ophthalmology, Ospedale Sant’Antonio, Azienda Ospedaliera, 35127 Padova, Italy; 7 Imperial College Ophthalmic Research Group (ICORG) Unit, Imperial College, 153-173 Marylebone Rd, London NW1 5QH, UK; 8 Department of Medicine and Surgery, University of Enna “Kore”, Piazza dell’Università, 94100 Enna, Italy; 9 Mediterranean Foundation “G.B. Morgagni”, 95125 Catania, Italy

**Keywords:** glaucoma, intraocular pressure, trabecular meshwork, Schlemm’s canal, stem cells, regenerative medicine

## Abstract

Glaucoma causes progressive damage to the optic nerve, and reducing intraocular pressure is the only clinically proven way to slow the disease. Although current medical, laser, and surgical treatments mainly change aqueous humor dynamics, they do not counteract the cellular and extracellular abnormalities of the conventional outflow pathway. Growing evidence shows that loss and dysfunction of trabecular meshwork cells impair aqueous humor drainage, supporting regenerative approaches that rebuild the outflow system rather than bypass it. In this narrative review, we present novel stem cell-based strategies to regenerate the trabecular meshwork and Schlemm’s canal, particularly trabecular meshwork (TM) stem cells, mesenchymal stem cells, pluripotent stem cell-derived TM-like cells, and neural crest-derived progenitors. We review recent evidence suggesting that transplanted cells may restore outflow not just through engraftment and cellular replacement, but also through paracrine signaling, stimulation of endogenous cell proliferation, extracellular matrix modification, and rejuvenation of the trabecular microenvironment. This review discusses advances in lineage specification, cell targeting and delivery, single-cell and transcriptomic characterization, mechanobiology, and bioengineered models of the human aqueous out-flow pathway. New approaches, such as magnetically guided cell delivery and the identification of cells that promote trabecular tissue regeneration and repopulation, show how rapidly this field has developed. We cover key translational barriers, including cell identity, manufacturing standardization, long-term engraftment, tumorigenicity, immune responses, off-target localization, and limited clinical efficacy data. Reconstructing the aqueous outflow pathway using stem cells remains a preclinical endeavor. However, it may shift the focus from chronic pressure control to biologically restoring outflow function in glaucoma.

## 1. Introduction

The term “glaucoma” refers to a large group of conditions that have in common a progressive loss of retinal ganglion cells and damage to the optic nerve, which ultimately leads to irreversible loss of vision. Its global burden is substantial and is expected to increase as populations age. A systematic review and meta-analysis including 50 population-based studies found that the global prevalence of glaucoma was estimated at 3.54% (95% credible interval [CrI], 2.09–5.82) among people aged 40–80 years. In 2013, there were an estimated 64.3 million affected people globally, and this figure is expected to rise to 76.0 million by 2020 and 111.8 million by 2040 [1]. Although glaucoma is a complex disease with many genes and environmental factors implicated in its pathogenesis, intraocular pressure (IOP) is, so far, the only modifiable risk factor whose reduction has been shown to slow glaucomatous visual loss in large clinical studies [2].

The randomized clinical evidence supports the connection between disease progression and IOP reduction. In the Early Manifest Glaucoma Trial, patients on laser trabeculoplasty and topical betaxolol had a mean IOP reduction of 5.1 mmHg, approximately 25% from baseline, maintained during a median follow-up of 6 years. Compared to untreated controls, disease progression occurred in 45% of treated patients versus 62% of untreated patients, and progression occurred significantly later in treated patients [3]. According to the United Kingdom Glaucoma Treatment Study, which demonstrated the effect of pharmacological pressure reduction in a placebo-controlled setting, at 24 months the mean IOP reduction was 3.8 mmHg with latanoprost compared with 0.9 mmHg with placebo. In addition, visual field preservation was significantly prolonged with treatment, with an adjusted hazard ratio of 0.44 (95% confidence interval, 0.28–0.69; *p* = 0.0003) [4].

The long-term Laser in Glaucoma and Ocular Hypertension (LiGHT) trial contributed evidence for the efficacy of an outflow-directed intervention. At the 6-year mark after selective laser trabeculoplasty (SLT), 69.8% of eyes were at or below target IOP without needing further medical or surgical treatment. Disease progression occurred in 19.6% of eyes first given SLT compared with 26.8% of those first given topical treatment (*p* = 0.006). Trabeculectomy was required in 13 versus 32 eyes (*p* < 0.001) [5]. Collectively, these data establish IOP reduction as the central therapeutic principle in glaucoma. Moreover, they establish an important distinction: presently effective interventions control the pressure-related component of the disease. However, restoring the biological integrity of the tissue responsible for physiological outflow is a different therapeutic objective.

Intraocular pressure (IOP) is determined by the balance between the production and the drainage of aqueous humor. Aqueous humor leaves the eye mainly via the conventional pathway, which includes the trabecular meshwork (TM) and Schlemm’s canal (SC), while some also leaves via unconventional pathways [2]. In the conventional route, the majority of aqueous outflow resistance originates within or in close proximity to the inner wall endothelium of SC. Although aqueous flow through this region is influenced by giant vacuoles and endothelial pores, resistance is considered more likely to arise from either the extracellular matrix (ECM) of the juxtacanalicular connective tissue or the basement membrane of SC [6]. This region is therefore not simply a passive anatomical filter. TM and SC cells contribute to a homeostatic mechanism that is adaptive in nature, where sustained elevations in IOP are sensed as mechanical stretch or distortion and are followed by adjustments in aqueous outflow resistance. Experimental evidence indicates that this response involves a complex process of ECM turnover occurring over hours to days, although the complete molecular mechanisms underlying IOP homeostasis remain incompletely defined [7].

Such a dynamic regulatory role is consistent with the cellular biology of the TM. TM cells are neural crest-derived cells having phenotypic and functional characteristics overlapping those of endothelial cells, fibroblasts, smooth muscle cells, and macrophages. Their functions include maintenance and remodeling of the surrounding matrix, generation of contractile forces, and phagocytic clearance of material entering the outflow pathway. As a result, during life, these cells are subjected to continuous mechanical, oxidative, metabolic, and phagocytic stress [8]. It is thus critical to maintain a sufficient number of functionally competent TM cells to maintain normal outflow physiology.

The loss of cells is a major aspect of TM aging. A study conducted on human samples spanning birth to 81 years through quantitative morphometric analysis revealed that TM cellularity and absolute cell number were reduced by 58% and 47%, respectively, across the examined age range. Regression analysis indicated a linear decline with age that corresponds to a loss of approximately 0.58% of TM cells per year [9]. However, the cellular abnormalities associated with primary open-angle glaucoma (POAG) are more complex than an acceleration of uniform age-related depletion.

In another analysis of 49 POAG and 69 nonglaucomatous specimens, TM cellularity was found to be lower in glaucomatous eyes across a wide variety of ages; however, both groups continued to exhibit age-related cell loss. Regional analysis revealed that the variation was mostly limited to the inner and intermediate TM. In contrast, the juxtacanalicular region’s cellularity was similar in glaucomatous and nonglaucomatous specimens. After 10 years of age, the expected rates of cellular decline varied between layers of the filtration region, with average rates of approximately 0.56% per year in the juxtacanalicular zone, 0.61% per year in the intermediate zone, and 0.69% per year in the inner TM [10]. Consequently, glaucomatous cellular loss is spatially heterogeneous and does not simply amount to a generalized depletion of the region that generates the majority of outflow resistance.

Cell loss in glaucoma occurs within an extracellular environment that is itself profoundly altered. Atomic force microscopy measurements demonstrated a significant difference in the mechanical properties of human trabecular meshwork tissues, with an average elastic modulus of 80.8 kPa in glaucomatous tissue as compared with 4.0 kPa in normal tissue. According to mathematical modeling, increasing TM stiffness leads to an increase in outflow resistance [11]. These biomechanical abnormalities are associated with ECM organization. The ECM found in the juxtacanalicular TM and SC basement membrane contributes importantly to the generation and regulation of outflow resistance, and its composition undergoes continual remodeling as part of IOP homeostasis [12]. The cell–matrix relationship becomes dysregulated in glaucoma. Alterations in ECM abundance, organization, cross-linking, and matrix-dependent signaling may heighten tissue rigidity and alter TM cell behavior, creating a feedback loop during which abnormal matrix properties influence the cells responsible for sustaining the same matrix [13]. The conventional outflow pathway in glaucoma, therefore, is better thought of as having a disturbed cellular and biomechanical microenvironment, rather than just an area of higher hydraulic resistance.

These observations provide the biological rationale for regenerative approaches. Current medical, laser, and surgical treatment methods for glaucoma use different strategies to lower IOP by reducing aqueous humor production or increasing its drainage; some act directly on the conventional outflow pathway, whereas others affect alternative components of aqueous humor dynamics [14]. According to clinical evidence, these techniques can yield a significant and long-lasting pressure-lowering effect, as shown in clinical studies [3,4,5], albeit not through replacement of cellular populations lost through aging and glaucoma. By contrast, stem cell-based approaches have been suggested for restoration of TM cellularity and functionality.

According to the reviewed literature, preclinical studies on tissue-specific stem cells, embryonic stem cells, induced pluripotent stem cells, and adult mesenchymal stem cells have shown restoration of TM cellularity and IOP homeostasis in experimental glaucoma and ex vivo models [14]. Nevertheless, the mechanistic nature of the link between TM cellularity and outflow resistance remains insufficiently resolved. Reduced cellularity is associated with impaired outflow in various experimental settings. However, a direct causal relationship between loss of specific TM populations and increased resistance has not been fully established [14]. Regenerative therapy should, therefore, be considered as not just replacement of missing cells, but an attempt to restore the functional cellular–matrix system responsible for physiological aqueous humor drainage.

For conceptual clarity, the regenerative cell populations considered in this review can be divided into several biologically distinct categories. Resident trabecular meshwork stem/progenitor cells (TMSCs) are tissue-associated populations isolated from the adult trabecular meshwork and characterized by combinations of stem-associated markers, clonogenic or self-renewal properties, and the capacity to acquire differentiated TM functions. Mesenchymal stem/stromal cells (MSCs) are multipotent adult cells obtained from extraocular tissues, such as bone marrow or adipose tissue, that may contribute to outflow repair either after TM-directed differentiation or through paracrine mechanisms.

Pluripotent stem cells, including induced pluripotent stem cells and embryonic stem cells (iPSCs), provide expandable populations that can be developmentally specified toward TM-like or other outflow-associated phenotypes. Neural crest-derived progenitors represent a developmentally informed strategy based on the embryological origin of anterior-segment tissues. These populations differ in developmental origin, differentiation potential, manufacturing requirements, and proposed mechanism of action and should therefore not be considered interchangeable forms of stem cell therapy.

This narrative review examines the biological and translational evidence supporting stem cell-mediated regeneration of the conventional aqueous outflow pathway in glaucoma. There is considerable interest in resident trabecular meshwork stem cells, mesenchymal stem cells, pluripotent stem cell-derived TM-like populations, and neural crest-derived progenitors, as well as the ways these could potentially restore TM and SC function. Moreover, strategies for cell specification, targeting, and delivery are considered. Most importantly, the influence of the extracellular and mechanical microenvironment on regenerative efficacy is noted. We aim to investigate whether new techniques can evolve from experimental outflow restoration to reproducible biological reconstruction of the conventional outflow pathway, and critically examine the barriers that currently separate these preclinical observations from clinical application.

Accordingly, the principal conceptual advance of this review is to integrate recent evidence indicating that outflow regeneration depends not only on the source of transplanted cells, but also on host–cell repopulation, regenerative cell state, paracrine signaling, targeted delivery, TM–SC cellular heterogeneity, and the mechanical properties of the recipient tissue.

**Figure 1:**
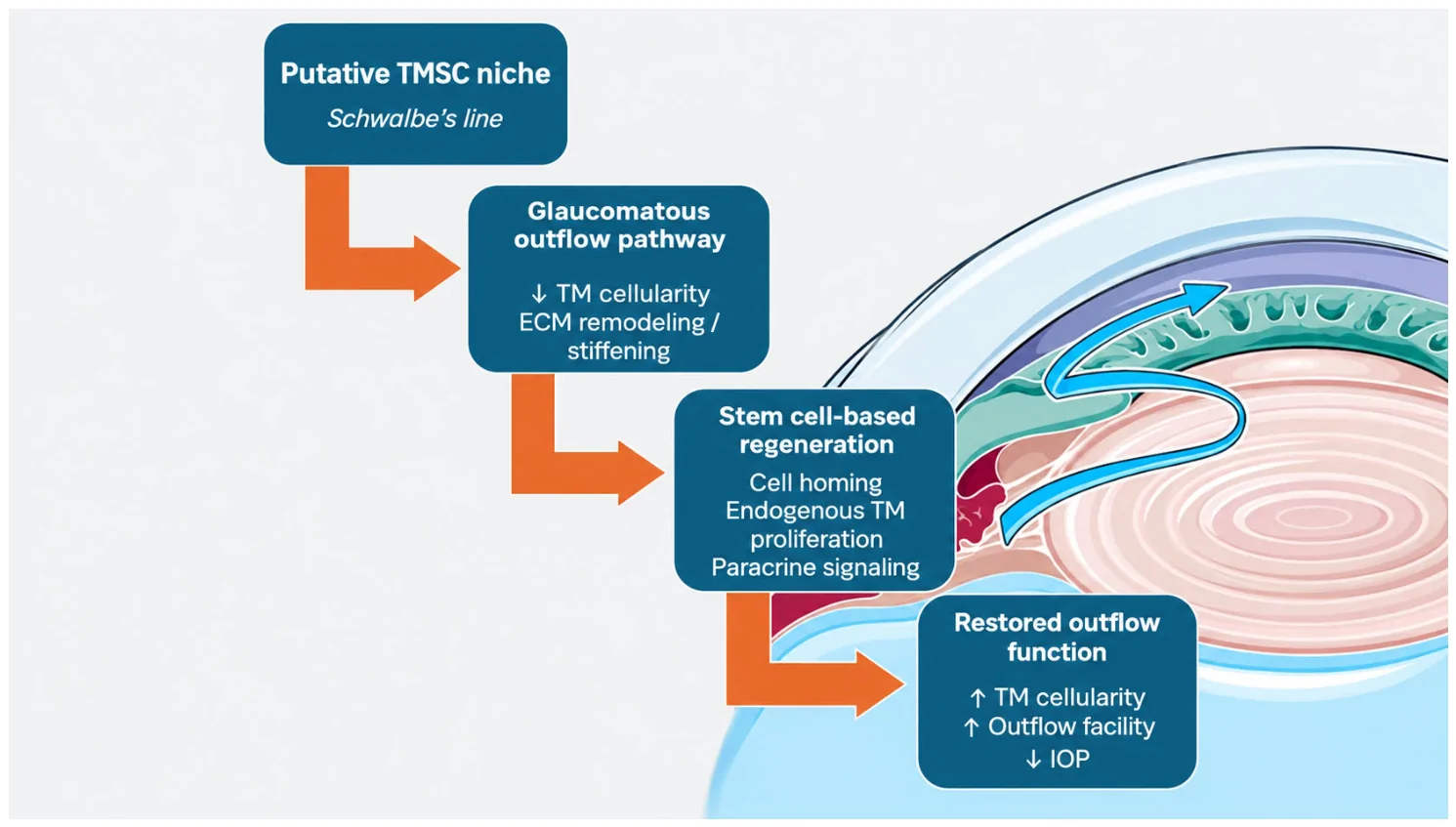
Conceptual framework of stem cell-mediated regeneration of the conventional aqueous outflow pathway in glaucoma. The glaucomatous conventional outflow pathway is characterized by reduced trabecular meshwork (TM) cellularity and pathological extracellular matrix (ECM) remodeling and stiffening. Putative trabecular meshwork stem cells (TMSCs) are concentrated near the Schwalbe’s line region. Stem cell-based regenerative strategies may act through homing and integration within the TM, stimulation of endogenous TM-cell proliferation, and paracrine signaling. These mechanisms may contribute to restoration of TM cellularity, improvement of conventional outflow facility, and reduction in intraocular pressure (IOP). The diagram is intended as a conceptual framework rather than an established linear causal sequence; the individual mechanisms shown are supported by different levels of experimental evidence. Images provided by Servier Medical Art (https://smart.servier.com), licensed under CC BY 4.0 (https://creativecommons.org/licenses/by/4.0/).

The principal structural abnormalities of the glaucomatous conventional outflow pathway and the proposed mechanisms through which stem cell-based approaches may restore outflow function are summarized in Figure 1.

## 2. Materials and Methods

The purpose of this study was to conduct a structured narrative review of the literature regarding stem cell-based regeneration of the conventional aqueous humor outflow pathway in glaucoma. The aim of the review, rather than being a systematic review or quantitative meta-analysis, was to provide a critical and mechanistically oriented synthesis of existing evidence.

Up to 22 September 2026, we updated our literature search using PubMed/MEDLINE and looked through the reference lists of pertinent original articles and key reviews to find other important or mechanistically relevant studies. Searches used combinations of the following terms: “glaucoma,” “primary open-angle glaucoma,” “ocular hypertension,” “aqueous humor outflow,” “trabecular meshwork,” “Schlemm’s canal,” “trabecular meshwork stem cells,” “trabecular meshwork progenitor cells,” “mesenchymal stem cells,” “adipose-derived stem cells,” “induced pluripotent stem cells,” “embryonic stem cells,” “neural crest cells,” “stem cell transplantation,” “regeneration,” “cell replacement,” “paracrine signaling,” “extracellular vesicles,” “exosomes,” “extracellular matrix,” “mechanobiology,” “matrix stiffness,” “single-cell RNA sequencing,” “transcriptomics,” “magnetic cell delivery,” “organ culture,” “three-dimensional models,” and “bioengineering.”

Studies were considered relevant when they directly investigated the trabecular meshwork, Schlemm’s canal, or the conventional aqueous outflow pathway in the context of cellular regeneration, stem or progenitor cell biology, tissue repair, or mechanisms possibly relevant to regenerative therapy. The first consideration was original studies involving human tissue or human anterior-segment models, experimental glaucoma or outflow-dysfunction models, and mechanistic in vitro studies. Primary literature was identified, and biological and translational context was provided using major reviews. Special emphasis was placed on studies published from 2019 to September 2026, whereas earlier studies were retained when they provided fundamental evidence related to TM cellularity, aqueous outflow resistance, neural crest origin, identification of stem/progenitor populations, or proof-of-concept cell transplantation.

Research solely aiming to replace retinal ganglion cells or neuroprotection—without any direct mechanistic link to regeneration of the conventional outflow pathway—fell outside the review’s main focus. When preliminary and subsequently peer-reviewed versions of the same study were available, the peer-reviewed version was used preferentially. Although preprints were not an emphasis, emerging studies without a peer-reviewed counterpart were considered if they closely matched the review’s scope, with reference to their non-peer-reviewed nature in the text.

The literature is very heterogeneous regarding cell source and phenotype, differentiation protocols, experimental species, glaucoma models, delivery strategies, follow-up duration, and functional outcomes, so quantitative pooling was not considered appropriate. The information was then put together based on five interconnected domains: cell source and identity, mechanisms of regeneration, target tissue, delivery strategy, and functional and translational relevance. Special care was taken to differentiate direct donor-cell replacement from stimulation of endogenous repair, as well as human tissue findings from those made based exclusively on animal or in vitro studies.

Because this was a structured narrative rather than systematic or scoping review a formal Preferred Reporting Items for Systematic Reviews and Meta-Analyses (PRISMA)-style screening log, and record-level count of screened and excluded studies were not prospectively maintained. The use of PubMed/MEDLINE as the primary database, supplemented by reference-list screening, may therefore have resulted in incomplete capture of the relevant literature and represents a limitation of the present review. This review did not involve the performance of any new human or animal experiments, nor the generation or analysis of new datasets.

## 3. Results: Current Evidence for Regeneration of the Conventional Aqueous Outflow Pathway

### 3.1. A Resident Regenerative Compartment Within the Trabecular Meshwork

The biological rationale for cellular regeneration of the conventional outflow pathway first required evidence that the adult human trabecular meshwork contains a population with properties distinct from those of differentiated TM cells. Stem-associated markers ABCG2, Notch1, OCT3/4, AnkG, and MUC1 were expressed by human TM-derived stem cells isolated either as side-population cells or via clonal expansion, while differentiated TM markers AQP1, MGP, CHI3L1, and TIMP3 were not expressed. After expansion, over 95% of the cells exhibited CD73, CD90, CD166, or Bmi1. Notably, these cells maintained their ability to differentiate into multiple lineages. When directed to take on a TM phenotype, they acquired expression of AQP1, CHI3L1, and TIMP3 along with phagocytic activity. This provided functional rather than exclusively phenotypic evidence of TM differentiation [15]. Another investigation identified an expandable TM-derived population expressing the mesenchymal markers CD73, CD90, and CD105 and capable of adipogenic, osteogenic, and chondrogenic differentiation. Its molecular profile showed considerable overlap with MSCs from other tissues, and it retained expression of genes characteristic of adult TM [16].

The regenerative contribution of these cells depends not only on their phenotype but also on their ability to recognize and incorporate into the outflow tissue. Following the administration of fluorescently labeled human TMSCs in the anterior chamber (AC) of normal mice, the TMSCs mostly localized to the TM region and were detectable for at least four months. TM-associated transplanted cells expressed CHI3L1 within one week. On the contrary, injected fibroblasts were more widely distributed in the TM, corneal endothelium, and lens epithelium and did not express CHI3L1. TMSC administration did not result in a detectable increase in IOP, apoptosis, accumulation of inflammatory cells, or local cell proliferation in otherwise normal eyes [17]. As a result of these findings, two properties ultimately became central to subsequent regenerative strategies: intrinsic tropism for the TM and the capacity to acquire a differentiated TM-like phenotype once within the tissue.

Later studies elaborated on the anatomical localization and quantitative extent of the presumed endogenous stem-cell compartment. In native human TM, the definition of TM stem cells was based on the combined presence of high ABCG2 expression and a nucleus-to-cytoplasm ratio greater than 0.7 rather than the expression of a single stem-cell marker. Cells that met both criteria also expressed p75 and AnkG. Using histological methods, cells with high ABCG2 and p75 expression were localized to Schwalbe’s line in the anterior non-filtering TM, while the filtering meshwork showed lower or minimal ABCG2 expression and was p75-negative [18].

The compartment showed a marked decline with age. The percentage of isolated native TM cells that were both high in ABCG2 expression and had a high nucleus-to-cytoplasm ratio was 21.0 ± 1.4% in donors younger than 30 years, 12.6 ± 6.6% in donors aged 30–60 years, and 4.0 ± 3.5% in donors older than 60 years, with a strong inverse correlation with age (rho = −0.88, *p* < 0.001) [18]. The decline was replicated in intact tissue sections. The total TM cellularity of donors younger than 30 years was 134 ± 30 cells per section. This decreased to 93 ± 16 in the 30–60-year group and further reduced to 80 ± 17 in donors older than 60 years (rho = −0.92, *p* = 0.0004). Over the same age range, the proportion of ABCG2-high cells declined from 9.4 ± 3.0% to 7.2 ± 4.0% and 5.4 ± 4.0%, respectively (rho = −0.79, *p* = 0.011). Similarly, the proportion of p75-positive cells declined from 11.2 ± 4.4% to 7.1 ± 6.0% and 0.3 ± 1.0%, respectively (rho = −0.81, *p* = 0.008) [18].

The age-associated decline in putative TMSCs paralleled the decline in total TM cellularity. Total cell number correlated strongly with the number of ABCG2-high cells (rho = 0.97, *p* < 0.001) and, to a lesser extent, with p75-positive cells (rho = 0.71, *p* = 0.031) [18]. While this association does not establish that stem-cell depletion causes age-related loss of TM cells, a limitation acknowledged by the authors, it provides evidence for the existence of an age-sensitive regenerative compartment whose reduction accompanies loss of the differentiated TM population.

A progenitor population that partially overlaps can be identified via p75 neurotrophin receptor expression. About 32% of isolated TM cells were p75-positive, but clonogenic cells appeared to be only a small subset, with a colony-forming efficiency of 1.11 ± 0.18%. The progenitors were negative at baseline for differentiated TM markers but expressed neural crest-associated factors including SOX9, SOX10, TFAP2B, FOXC1, and FOXC2. Subsequent to directed differentiation, they developed expression of AQP1, MGP, prostaglandin D2 synthase, and AnkG, while exposure to dexamethasone increased expression of myocilin [19]. The findings support the idea that marker expression alone does not fully define the regenerative compartment and that stem/progenitor properties are distributed among partially overlapping TM populations.

These observations also highlight an unresolved nomenclature problem. Putative TMSCs, TM progenitor-like cells, and expanded TM-derived mesenchymal-like populations should not be assumed to represent the same biological entity. No universally accepted minimum definition of a bona fide adult TMSC currently exists. Marker expression alone is insufficient, particularly because ABCG2, p75, mesenchymal markers, clonogenicity, label retention, sphere formation, and nucleus-to-cytoplasm ratio identify partially overlapping rather than identical populations. A more rigorous designation would therefore require convergent evidence of a stem/progenitor phenotype, self-renewal or clonogenic capacity, acquisition of functional TM characteristics after differentiation, and appropriate tissue localization or regenerative behavior. Until such criteria are standardized, populations defined primarily by marker expression are more appropriately described as putative TMSCs or TM stem/progenitor-like cells.

Functional assays provide further evidence of this heterogeneity. ABCG2/p75-positive adherent cells in cultured human TM represented 0.96 ± 0.21% of all cells from donors younger than 30 years, compared to 0.32 ± 0.23% of all cells from donors aged 30–60 years and 0.35 ± 0.04% of all cells from donors older than 60 years. About 7.00 ± 1.80% of cultured cells were label-retaining, and sphere-forming efficiency was 0.82 ± 0.23%. After dexamethasone exposure, 86.00 ± 14.87% of sphere-derived cells were myocilin-positive, and 64.60 ± 7.24% developed cross-linked actin networks. Their phagocytic activity was 13.28 ± 3.30% versus 16.33 ± 4.04% in primary TM cells [20]. Clonogenicity, label retention, expression of stem-associated markers, and functional differentiation reveal overlapping but non-identical subsets of the adult human TM.

Subsequently, donor tissue was used to directly investigate the relevance of this regenerative compartment to glaucoma. The overall TM cellularity in POAG eyes was reduced to 41.83 ± 9.00 cells per section as compared to 69.33 ± 12.77 cells per section in age-matched nonglaucomatous controls (*p* = 0.0007). The percentage of cells that coexpressed high levels of ABCG2 and p75 was also numerically lower in glaucomatous eyes, decreasing from 4.73 ± 5.46% in controls to 0.14 ± 0.17% in POAG specimens, although this difference did not reach statistical significance (*p* = 0.064) [21]. These cellular changes were accompanied by structural alterations, with the trabecular beams becoming thicker and fused together, along with decreases in TM and Schlemm’s canal dimensions. Thus, it is firmly established by the available human evidence that there is a loss of overall TM cellularity in glaucoma and an age-associated depletion of putative TMSCs, while the extent to which glaucoma independently accelerates depletion of the stem/progenitor compartment is less certain.

### 3.2. From Homing to Functional Tissue Repair

After establishing a regenerative population, the following requirement is durable localization within the diseased tissue. Cell retention appears to partially depend on interactions of the transplanted cells with the extracellular matrix. Human TMSCs express higher levels of α5β1 integrin as compared to fibroblasts. In addition, blockade of α5β1 lowered the attachment of TMSCs to TM cells and fibronectin in vitro. Even though untreated TMSCs and TMSCs in which α5β1 had been blocked both reached the mouse TM after intracameral injection, cells in which α5β1 had been blocked were no longer retained at one month, indicating that homing and durable integration are mechanistically separable processes [22].

Injury seems to strengthen the signals that guide localization. In a mouse model in which 180° of the TM was damaged by laser photocoagulation, transplanted human TMSCs preferentially accumulated in laser-treated regions and expressed differentiated TM markers at two and four weeks. Following transplantation, the laser-induced inflammatory and fibrotic responses decreased, and the TM’s ultrastructure and outflow function were restored. The researchers found that CXCR4 expression increased in TMSCs and SDF1 increased in the damaged TM. Migration and affinity assays supported the involvement of the CXCR4/SDF1 axis in injury-directed homing [23]. Thus, the diseased microenvironment not only serves as a structural target but also modifies the signals regulating recruitment and retention of cells.

Functional rescue has been demonstrated most extensively in the Tg-MYOC^Y437H model. At four months of age, untreated transgenic mice had a mean IOP of 16.50 ± 0.44 mmHg compared with 12.38 ± 0.41 mmHg in wild-type animals (*p* < 0.0001). One month after intracameral TMSC transplantation, the IOP dropped to 13.30 ± 0.42 mmHg, close to that of wild-type animals (12.30 ± 0.45 mmHg), and remained significantly less than that of untreated transgenic mice. An increase in conventional outflow facility and restoration of TM cellularity was observed. The regenerated tissue showed improved ultrastructure and ECM organization, secretion of mutant myocilin into the aqueous humor increased, and intracellular endoplasmic-reticulum stress was reduced. Retinal ganglion cell survival and function were improved in transplanted eyes [24]. The significance of these findings lies in the fact that they associate cellular repair of the outflow tissue with restoration of its hydrodynamic function rather than relying on IOP reduction alone as a surrogate of regeneration.

Human anterior-segment organ culture has provided supporting evidence in a direct cell-loss model. Saponin treatment induced TM cell death (32.08 ± 4.74%) and reduced outflow facility by 15% by day 6. The introduction of 3 × 10^5^ cultured human TMSCs increased outflow by 38% compared to the cell-loss state and resulted in homing to filtering and non-filtering TM regions. Ki67-positive TM-cell proliferation was 17.94 ± 4.45% five days after transplantation compared with 5.29 ± 4.20% after sham treatment [25]. The magnitude of endogenous proliferation in relation to the transplanted population is significant, as it points away from a purely stoichiometric replacement model.

### 3.3. Paracrine Signaling as a Component of TMSC-Mediated Regeneration

Recent studies suggest TMSC-mediated repair can take place by modulating surviving TM cells. Following exposure to transforming growth factor beta 2 (TGF-β2), TMSC resistance was significantly higher than that of differentiated TM cells to the loss of viability, mitochondrial depolarization, generation of reactive oxygen species, lipid peroxidation, and induction of fibrotic markers. TMSCs and their secretome were found to have enriched hepatocyte growth factor (HGF), as identified by RNA sequencing and cytokine profiling. The TMSC-conditioned secretome and recombinant HGF both reduced the oxidative and fibrotic responses in TM cells. Further pharmacological inhibition of the HGF receptor caused a reduction in this protective effect. Notably, inhibition of HGF did not block the whole effect, indicating that HGF is one of the components but not the only mediator of the TMSC secretome [26]. In this Review, “extracellular vesicle” is used as the general term for released membrane-bound vesicles, whereas “exosome” is reserved for studies in which an endosomal origin or an appropriately characterized exosome-enriched population was reported.

Small extracellular vesicles represent an additional mechanism whereby the regenerative signal may be transferred without lasting donor-cell integration. Small extracellular vesicles derived from TMSCs were measured to be approximately 30–200 nm and expressed the exosomal marker syntenin together with TM-associated vesicular proteins. In vitro studies showed that they improved scratch-wound closure and proliferation and migration of TM cells. In addition, they increased cell survival under chronic oxidative stress while reducing intracellular reactive oxygen species [27]. These experimental results are in vitro and therefore do not establish that extracellular vesicles alone are sufficient for restoring outflow in vivo. However, they supplement data on transplantation by showing that the release of biologically active information from TMSCs can directly affect the behavior of differentiated TM cells.

Taken together, the studies presented in [22,23,24,25,26,27] support a continuum and not a dichotomy between cell replacement and paracrine regeneration. TMSCs can reach and integrate into the TM. However, restoration of tissue cellularity and function also involves recruitment or proliferation of endogenous cells and modification of the local stress and ECM environment.

### 3.4. Mesenchymal Stem Cells and Adipose-Derived Cells as Alternative Regenerative Sources

Because regeneration can be mediated partly by the action of secreted factors, interest has now extended beyond tissue-resident TMSCs. In a laser-induced ocular hypertension model, tissue repair and intraocular pressure reduction were superior with bone marrow-derived mesenchymal stem cells compared with hematopoietic cells. The conditioned medium taken from MSCs that had been exposed to low-oxygen conditions produced an immediate IOP-lowering effect, but the medium from cells maintained under normal oxygen conditions did not. The MSC approach and secreted factors were also associated with reactivation of a progenitor population in the ciliary body and persistent proliferating cells at the anterior-chamber angle for at least a month [28]. The study was among the first to experimentally show that stem-cell-mediated repair of the outflow pathway need not require the administered cells to differentiate into mature TM cells.

MSCs and TM cells are not interchangeable despite this mechanistic flexibility. A direct comparison of their molecules, proteins, and functions indicated substantial differences between adipose-derived MSCs and primary TM cells. According to the researchers, 12 transcripts were required to identify cell identity and differentiation state, whereas dexamethasone-induced myocilin represented a robust protein-level discriminator. The two populations also exhibited differences in contractile and phagocytic behavior [29]. The expression of a limited number of overlapping mesenchymal markers has been found to be insufficient to establish acquisition of a functional TM phenotype.

Adipose-derived stem cells (ADSCs) nevertheless provide a potentially scalable cell source. Following induction toward TM-like differentiation, ADSC-derived TM-like (ADSC-TM) cells acquired a transcriptional profile associated with TM, phagocytic function, and dexamethasone responsiveness. After administration in normal mice, both undifferentiated ADSCs and differentiated ADSC-TM cells localized to the TM, expressed TM-associated markers, and did not lead to changes in normal IOP, outflow facility, or ECM organization. Migration and adhesion experiments again implicated the CXCR4/SDF1 signaling system in TM targeting [30]. The experiments indicate compatibility with normal outflow tissue and not the rescue of established glaucoma. Their interpretation should thus be kept distinct from therapeutic studies in diseased models.

Researchers explored the cell-free part of the MSC mechanism directly. Human bone-marrow MSC-derived exosomes, with a mean diameter of about 130 nm, were progressively internalized by human TM cells over 6–24 h. According to a study [31], pretreatment of TM cells protected them from H_2_O_2_-induced injury. Furthermore, there were reductions in oxidative-stress and inflammatory responses. Most importantly, changes were observed in matrix metalloproteinase expression. Another study found that MSC-derived exosomes enhanced TM-cell phagocytosis, reduced cell damage, and modified NRF2-related oxidative-stress signaling [32].

Given all the preceding evidence, two conceptually distinct MSC-based routes are supported. The first route is differentiation into TM-compatible cells. The other route is the delivery of regenerative signals without terminal TM differentiation. The latter may ultimately prove particularly relevant if durable donor-cell replacement is not required for sustained restoration of tissue function.

### 3.5. iPSC-Derived Cells Reveal That Endogenous Repopulation Can Dominate over Donor-Cell Replacement

Pluripotent stem cells offer a different strategy because the starting population can be extensively expanded before specification toward an outflow phenotype. The first differentiation of iPSCs through coculture with human TM cells produced cells that progressively lost expression of the pluripotency markers Nanog, Oct4, and Sox2. They began to exhibit TM-like morphology and other molecular characteristics. Functionally, differentiated cells became phagocytic and responded to dexamethasone with increased myocilin production and to phorbol 12-myristate acetate with increased matrix metalloproteinase-3 secretion [33]. As a result of these experiments, several independent TM-associated responses were acquired, rather than expression of a single marker.

Researchers conducted a test to determine the functional relevance of cellular replenishment through experimental TM cellular depletion. The disturbance of normal IOP homeostasis occurred due to loss of TM cellularity. Transplantation of iPSC-derived TM-like cells repopulated the tissue, and restoration of pressure regulation in response to perfusion was observed [34]. This supplied evidence that cellular depletion can itself impair outflow homeostasis and that a stem-cell-derived population can restore the regulatory response.

In vivo studies subsequently showed that the mechanism is not simply replacement of each missing TM cell by a transplanted cell. In the Tg-MYOC^Y437H mouse model, iPSC-TM transplantation lowered IOP and increased outflow facility for at least nine weeks. Moreover, there was also preservation of retinal ganglion cells. TM cellularity increased markedly, but studies found that the increase originated from proliferation of the TM cell population already present in the experimental system rather than expansion of the transplanted cells [35]. The same principle was true in older, six-month-old transgenic mice with more advanced TM dysfunction. Twelve weeks post-transplantation, IOP was reduced and outflow facility was increased compared with untreated animals, while endogenous TM cellularity increased with the appearance of mitotically active TM cells. Even though myocilin and calnexin remained elevated, indicating that molecular endoplasmic-reticulum (ER) stress persisted despite functional rescue [36], TM ultrastructure and endoplasmic-reticulum morphology were better preserved.

This endogenous response was then assessed in perfused anterior segments obtained from aged human donors. In the regenerated TM tissue, fewer than 1% of cells were derived from the transplanted iPSC-TM. However, the cellularity of the TM was significantly increased at 7 and 14 days after iPSC-TM transplantation. In addition, Ki67 positivity and bromodeoxyuridine (BrdU) incorporation in endogenous cells increased [37]. This finding lies at the heart of the regenerative model: a small number of donor cells can elicit a larger host–cell response.

Experiments aimed at identifying a mechanism found direct intercellular communication to be one component of this response. In a mouse model of glaucoma, using pharmacological or genetic techniques to inhibit connexin-43-dependent gap-junction communication reduced the ability of iPSC-TM cells to stimulate endogenous TM-cell proliferation. Calcium transfer from donor to host cells was implicated in this signaling [38]. For this reason, endogenous repopulation is not just temporally linked to transplantation, but it can also be associated with specific donor–host communication pathways.

### 3.6. Developmentally Guided Specification and Regenerative Cell States

The phenotype produced from pluripotent cells also depends on the developmental pathway used for differentiation. A two-step approach first directed the cells toward neural crest-like cells and then exposed them to TM-cell-derived ECM. The resulting cells acquired TM-associated morphology and molecular characteristics, responded to dexamethasone with increased myocilin, and formed cross-linked actin networks [39]. This method is more similar to the developmental origin of TM than direct phenotype induction.

More recent work in multi-modal transcriptomics suggests that the most therapeutically relevant derivative may not be the population that most resembles a mature TM cell. iPSC differentiation generated a population of ITGA6+ cells with a transcriptional profile distinct from primary TM cells. The iPSC-ITGA6+ cells were more effective than other iPSC-TM subpopulations in stimulating the proliferation and rejuvenation of primary TM cells and repopulation of TM and Schlemm’s canal. The effect was linked to increased NEAT1 transcription and paraspeckle abundance within the ITGA6-positive cells. Experimental increase in paraspeckle assembly through the use of MENβ-associated RNA further stimulated primary TM-cell proliferation and rejuvenation [40]. This finding is noteworthy because it distinguishes between two therapeutic properties that have commonly been considered synonymous: resemblance to the mature TM and regeneration-inducing potential.

Classical human evidence lends developmental justification to neural crest-based strategies. In normal adult eyes, cells of the anterior TM and inner uveal beams were found to express neuronal-specific enolase. Cultured TM cells progressively lost this expression after 18–21 days in vitro, but exposure to nerve growth factor restored it. The phenotype described in this paper was interpreted as evidence that human TM cells are derived from the embryonic neural crest [41].

Recently, scientists have used human embryonic stem cell (hESC)-derived neural crest cells for direct transplantation. After five days of chemically defined induction, HNK-1, p75, SOX10, and AP-2α expression were observed. Injection of 1.0 × 10^6^ cells with 100 μmol/L Y-27632 into rabbit anterior chambers resulted in integration within TM tissue along with expression of Aqp1, Chi3l1, and Timp3 seven days after transplantation. IOP and central corneal thickness remained within normal limits during the first two weeks, and no significant adverse effects were reported throughout five weeks of follow-up. Separate implantation into non-obese diabetic/severe combined immunodeficient (NOD/SCID) mice did not generate teratomas [42]. These findings establish short-term in vivo lineage adaptation and initial safety in this experimental paradigm, but therapeutic rescue of glaucomatous outflow dysfunction has not yet been demonstrated.

### 3.7. Targeted Delivery as a Determinant of Regenerative Efficiency

The fraction of all the cells injected that actually reaches the TM determines the efficacy of any cellular product. Passive delivery into the anterior chamber exposes cells to the entire anterior segment and results in inconsistent targeting. To respond to this constraint, magnetic steering was created. Magnetic targeting of the TM with mesenchymal stem cells (MSCs) labeled with Prussian blue nanocubes, within 15 min of magnetic exposure, showed greater TM delivery than unlabeled cells. The MSCs encircled the TM instead of remaining confined to the site of passive delivery. Labeling with nanoparticles had no measurable effect on the viability or multipotency of MSCs [43].

Substituting superparamagnetic iron oxide nanoparticles and optimizing the external magnetic geometry still further increased localization. A concentrated “point magnet” attracted more labeled MSCs toward the TM with more uniform circumferential distribution than unlabeled cells and the previous ring-magnet configuration [44]. The experiments showed that the efficiency of delivery may be modulated experimentally rather than being an intrinsic limitation of intracameral cell therapy.

The long-term study afterward demonstrated the functional consequences. A single administration of only 1500 magnetically steered human adipose-derived MSCs resulted in a median IOP decrease of 4.5 mmHg (interquartile interval reported by the authors, 3.1–6.0 mmHg), which corresponds to a 27% drop, and the effect was maintained for nine months. The decline was linked to increased conventional outflow and enhanced TM cellularity. The use of magnetically delivered iPSC-TM cells provided a reduction in IOP of 1.9 mmHg (0.4–3.3 mmHg), or about 13%. However, the iPSC-TM cells were associated with greater tumorigenicity concern. Cells that were delivered could still be located in the iridocorneal angle even after three weeks. However, their persistence was very limited when compared with the duration of the pressure effect. Therefore, the authors of the study favored a paracrine mechanism [45]. According to these data, targeting efficiency and biological potency are linked. Prolonged functional effects were obtained with a cell dose much smaller than those used in many earlier intracameral transplantation experiments.

The principal cell-based approaches investigated for regeneration of the conventional outflow pathway, together with their experimental models, functional outcomes, and major translational limitations, are summarized in Table 1.

### 3.8. Single-Cell Analyses Redefine the Cellular Target of Regeneration

A major limitation of early regenerative studies was that “TM-like” differentiation was assessed against a tissue treated largely as a single cellular entity. Single-cell transcriptomics indicates extensive heterogeneity of the conventional outflow pathway. Transcriptome analysis of around 24,000 single cells from human outflow tissues identified 19 cell types. Experiments conducted with cynomolgus and rhesus macaques, pigs, and mice revealed broad conservation between species, but with important differences in cellular composition and gene expression [46]. Understanding the implications is especially relevant in regenerative research since successful engraftment in a mouse TM does not guarantee molecular equivalence to the relevant human target population.

The independent profiling of human conventional outflow tissue identified numerous TM, Schlemm’s canal, vascular, immune, and nearby stromal populations and further demonstrated distinct transcriptional states within anatomically contiguous tissues [47]. As such, regeneration of the conventional pathway should be considered restoration of a multicellular system rather than production of a generic TM phenotype.

Schlemm’s canal follows the same principle. An analysis of single-cell gene expression demonstrated that SC endothelial cells are molecularly distinct from conventional blood vascular endothelium and overall biased toward a lymphatic-like identity. Notably, SC itself was heterogeneous: three major transcriptional states were observed, including distinct inner-wall populations, and ligand–receptor analyses predicted extensive signaling between SC and neighboring TM populations [48]. These observations offer a molecular rationale for considering the TM–SC interface as a coordinated functional unit.

A recent single-cell developmental study analyzed approximately 130,000 cells across stages of mouse anterior-segment development and reconstructed signaling interactions and candidate developmental trajectories involved in the formation of the TM and SC [49]. An overview of SC development integrates these developmental pathways with the current understanding of canalogenesis and their possible relevance to glaucoma treatment [50].

### 3.9. Schlemm’s Canal Is an Active Regenerative Target, Not Merely the Downstream Recipient of TM Repair

Developmental studies establish that SC is not a conventional venous channel. Evidence from lineage tracing indicates that SC forms after birth, and while its cells originate from blood vascular endothelial cells, they subsequently acquire a partial lymphatic program. This is the result of a different developmental process called canalogenesis [51]. This hybrid phenotype is strongly associated with prospero homeobox 1 (PROX1). During the course of development, SC endothelial cells acquire PROX1 along with certain lymphatic markers, while reduced aqueous humor flow results in loss of PROX1 and loss of canal identity. The expression of PROX1 was related to the structure and function of SC across experimental conditions [52].

An important distinction is required between endogenous regenerative capacity of the SC and the generation of SC-like cells from exogenous stem-cell sources. Unlike the TM, in which resident stem/progenitor populations have been isolated and characterized, a bona fide adult SC stem/progenitor population with established self-renewal and lineage-reconstitution capacity has not yet been clearly defined [50]. Evidence for endogenous SC regeneration currently relates primarily to plasticity or reactivation of developmental programs within existing endothelial populations. By contrast, exogenous stem cells can be experimentally directed toward an SC-like endothelial phenotype. For example, human adipose-derived stem cells exposed to vascular endothelial growth factor C (VEGF-C), fluidic shear stress, and cues from a bioengineered TM microenvironment acquired SC-associated characteristics, including PROX1 and vascular endothelial growth factor receptor 3 (VEGFR3) expression, and reproduced selected functional properties of primary SC endothelial cells in vitro [53]. These cells should therefore be regarded as stem-cell-derived SC-like endothelial cells rather than resident SC stem cells.

Even after development, the canal is molecularly plastic. The disruption of either Angpt1/Angpt2 or their receptor Tie2 in adult mice caused severe impairment of SC structure and transcytotic function. It also elevated IOP and caused retinal neuronal injury. Finally, it disrupted retinal ganglion cell function. On the other hand, drug-induced activation of Tie2 rescued the phenotype in Angpt1/Angpt2-deficient animals and induced structural rejuvenation of SC in aged mice [54]. Genetic evidence in humans provides support for the same developmental axis. The deletion of Angpt1 in mice led to a severely hypomorphic SC and elevated IOP. In contrast, simultaneous absence of Angpt1 and Angpt2 completely eliminated the canal. Three patients had rare ANGPT1 variants across an international cohort encompassing 284 patients with primary congenital glaucoma. Of the three alleles, functional evaluation supported loss of function for two variants [55].

More recent experiments identified macrophages as an additional component of SC morphogenesis. The vitronectin that is produced by limbal macrophages activates the αvβ3 integrin of the endothelium to promote signaling through AKT/FOXO1/β-catenin/FOXC2 and induces expression of PROX1. When this pathway’s function was genetically disrupted, ocular hypertension resulted from impaired canal formation, while aged SC regrew after FOXO1 inhibition [56].

The data on human disease support the relevance to glaucoma. Genome-wide expression profiling of primary SC cells from three glaucoma cases and four controls identified 113 transcripts showing at least twofold differential expression. Enriched pathways involved cell adhesion, heparin and glycosaminoglycan binding, filopodia, and ECM remodeling. Furthermore, 18 selected differentially expressed genes were independently validated by droplet digital polymerase chain reaction (PCR) [57]. Through analysis of their phenotype, it was determined that glaucomatous SC endothelial cells expressed increased levels of alpha-smooth muscle actin (α-SMA), collagen Iα1, fibronectin, and TGF-β2 compared to healthy SC cells. They were larger and showed enhanced proliferation and migration but reduced mitochondrial activity [58]. In this way, a regenerated TM would interface with an SC endothelium that might itself remain transcriptionally, metabolically, and mechanically abnormal.

These findings broaden the regenerative target from TM cellularity alone to the TM–SC unit. Direct stem-cell reconstruction of Schlemm’s canal remains substantially less developed than TM regeneration, and most current evidence for SC repair concerns endothelial plasticity, developmental-pathway reactivation, or pharmacological rejuvenation rather than direct stem-cell-mediated replacement [40].

### 3.10. Mechanobiology Defines the Environment in Which Regeneration Must Occur

Cell identity and delivery are only part of the regenerative problem because donor and endogenous cells operate within an abnormal glaucomatous extracellular matrix. In primary human TM cells cultured on biomimetic substrates, increasing ECM stiffness promoted nuclear localization of Yes-associated protein (YAP) and transcriptional coactivator with PDZ-binding motif (TAZ) and altered focal-adhesion and cytoskeletal organization. TGF-β2 independently increased nuclear YAP/TAZ in both normal and glaucomatous TM cells, and this response was reduced by inhibition of extracellular signal-regulated kinase (ERK) and Rho-associated kinase (ROCK) signaling [59]. Mechanical and profibrotic signals therefore converge on common mechanotransduction pathways capable of altering TM-cell phenotype.

A similar relationship is present in Schlemm’s canal. Dynamic hydrogel experiments showed that substrate stiffening induced YAP/TAZ activation and cytoskeletal reorganization in primary human SC cells, whereas subsequent matrix softening reversed these abnormalities over time. Pharmacological or genetic inhibition of YAP/TAZ attenuated stiffness-induced cytoskeletal and ECM changes. In ex vivo mouse eyes, perfusion with verteporfin without light activation increased conventional outflow facility [60]. These findings indicate that an established pathological mechanical phenotype is potentially reversible.

The stiffness of the matrix provokes distinct responses from normal and glaucomatous TM cells. When normal high-flow TM/juxtacanalicular connective tissue (JCT) cells were seeded onto collagen substrates with elastic moduli of 4.7 and 27.7 kPa, they generated more traction force on the stiffer matrix. In contrast, glaucomatous high-flow cells generated greater traction forces on the softer substrate. Normal cells are about 1.6 times stiffer than glaucomatous cells, which show altered cytoskeletal properties and reorganization of collagen [61]. The glaucomatous environment can therefore modify cellular behavior in directions not predicted based on the properties of the transplanted cell alone.

Bioengineered extracellular matrices, hydrogels, cell-derived matrices, organ culture, and three-dimensional outflow models have consequently become important intermediate platforms for evaluating these cell–matrix interactions under controlled mechanical conditions [62,63]. Because standard 2D culture cannot recreate the stiffness gradients, ECM organization, pressure loading, and multicellular interactions that transplanted cells experience in the outflow pathway, they are especially relevant for regeneration studies.

Finally, pharmacological remodeling provides evidence that the pathological outflow microenvironment can itself be functionally modified. In ocular hypertension caused by glucocorticoids, netarsudil prevented or reduced IOP elevation and attenuated fibrosis and tissue stiffening in the conventional pathway. In the preventive mouse trial, eyes treated with placebo experienced a mean rise in IOP of 5.94 ± 0.57 mmHg, while eyes treated with netarsudil showed no rise from baseline (0.23 ± 0.45 mmHg). The outflow facility in netarsudil-treated eyes was 4.87 ± 1.09 nL/min/mmHg, whereas in placebo-treated eyes it was 2.64 ± 0.44 nL/min/mmHg, representing an 84% difference, although reportedly not reaching conventional statistical significance (*p* = 0.08) [64].

Collectively, these findings indicate that regeneration of the conventional outflow pathway involves multiple interconnected cellular, paracrine, developmental, and biomechanical processes rather than a single mechanism of donor-cell replacement. The principal regenerative mechanisms and tissue-level targets emerging from the available evidence are summarized in Table 2.

## 4. Discussion

The evidence reviewed here supports a concept of glaucoma regeneration that is much broader than replacement of lost trabecular meshwork cells. In various experimental systems, regeneration of the conventional outflow pathway results from the interaction of transplanted cells, surviving endogenous cells, extracellular matrix, and Schlemm’s canal, rather than solely from repopulation with donor cells. The distinction is critical as it alters both the biological goal of therapeutic treatment and the benchmark against which a regenerative cell product should be evaluated. A cell that persists in the TM and acquires a mature phenotype may directly contribute to tissue replacement. However, a cell that has only limited long-term engraftment may still be therapeutically relevant if it activates endogenous repair, modifies the extracellular environment, or restores intercellular signaling.

The existence of an endogenous regenerative compartment provides a biological basis for this model, but its significance should not be overstated. Human TMSCs can be detected in the anterior non-filtering TM, primarily in the vicinity of Schwalbe’s line, although their number declines significantly with age alongside overall TM cellularity [18]. Nevertheless, the link between the two processes does not imply that TMSC depletion is causative of the age-related loss of differentiated TM cells. In addition, it was noted that, despite POAG donor tissue exhibiting an overall decrease in TM cellularity, the reduction in the ABCG2-high/p75-positive population was not statistically significant in the available glaucomatous sample [21]. The current evidence therefore supports an age-sensitive resident regenerative compartment and marked cellular depletion in glaucoma, though it does not yet establish failure of that compartment as a trigger of POAG. This distinction matters therapeutically, as regeneration may compensate for an established cellular deficit without necessarily indicating that stem-cell depletion is the primary pathogenic event. Among the cell populations considered in this review, TMSCs therefore represent the most tissue-specific candidate for biological restoration of the TM. Their potential advantage lies not only in their ability to acquire TM-associated phenotypic and functional characteristics, but also in their intrinsic localization and homing behavior within the conventional outflow pathway and their capacity to influence surviving endogenous TM cells. Nevertheless, the term TMSC currently encompasses partially overlapping populations identified by different combinations of stem-associated markers, clonogenicity, label retention, sphere formation, and differentiation capacity. Establishing a reproducible molecular and functional definition of TMSCs, together with scalable isolation and manufacturing criteria, will therefore be essential before their therapeutic potential can be meaningfully translated.

The increasing separation between donor-cell persistence and tissue regeneration is the strongest mechanistic convergence across the transplantation literature. TMSCs have shown the potential to migrate to damaged TM, integrate into the tissue, and support recovery of outflow function [22,23,24,25]. However, several other findings indicate that replacement alone cannot account for the whole response. This is especially the case with iPSC-derived TM cells, as increased TM cellularity in glaucomatous mice and perfused human eyes was driven mainly by proliferation of endogenous cells, with very few transplanted cells within the repopulated tissue [35,36,37]. One mechanism of donor cells promoting host–cell proliferation that is supported by experiments is connexin-43-dependent intercellular communication and calcium transfer [38]. Along the same lines, TMSC-derived HGF-containing secretome and small extracellular vesicles can modify survival, oxidative stress, proliferation, and migration of differentiated TM cells without requiring the actual conversion of the therapeutic cells themselves into mature TM [26,27]. MSC-conditioned products and MSC-derived exosomes have similar paracrine effects [28,31,32]. On the whole, these data support a model in which the therapeutic unit is not necessarily the engrafted donor cell itself but the regenerative response the donor cell can induce in the host tissue.

This distinction also challenges the assumption that the most appropriate therapeutic cell must be the one that most closely resembles a terminally differentiated TM cell. Particularly informative is the identification of an ITGA6-positive iPSC-derived population that has a transcriptional state distinct from primary TM cells but greater capacity to induce endogenous TM proliferation and repopulation of the conventional outflow pathway [40]. In this context, NEAT1 transcription and paraspeckle biology, rather than increasing molecular similarity to mature TM, were associated with regenerative potency. Developmentally guided differentiation through a neural crest intermediate, as shown in [39], and the integration of hESC-derived neural crest cells into rabbit TM and subsequent expression of mature TM markers, as stated in [42], suggest that differentiation stage and regenerative activity should be considered separately. Consequently, a transient progenitor-like or signaling-competent state may represent the most suitable therapeutic phenotype rather than a mature TM surrogate. Before this concept can be applied to cell-selection criteria, direct head-to-head comparisons will be necessary.

This question of cell identity has become more complex as the target tissue itself has been molecularly resolved. Research involving single-cell techniques has shown that the conventional outflow pathway comprises various distinct TM, Schlemm’s canal, vascular, immune, and stromal populations rather than a single population of “outflow cells” [46,47,48]. As a result, the presence of a small number of conventional TM markers does not by itself establish biological equivalence to the native therapeutic target. Future definitions of regenerative potency may need to incorporate molecular state together with functional properties such as phagocytosis, mechanosensitivity, ECM regulation, response to relevant stimuli, and communication with neighboring outflow populations. This is especially true when comparing TMSCs, MSC-derived products, iPSC derivatives, and neural crest-derived progenitors, since similar functional effects can be generated from biologically different cell states.

A related implication is that TM regeneration should not be considered independently of Schlemm’s canal. SC has a specialized endothelial identity that combines features of the blood and lymphatic vasculature and relies on tightly regulated developmental programs including PROX1 and ANGPT/Tie2 signaling [51,52,53,54,55]. Experimental manipulation of these pathways can alter or restore SC architecture after development, while macrophage-derived vitronectin–integrin signaling provides further evidence that canal identity is responsive to its cellular surroundings [56]. Human glaucomatous SC cells show transcriptional, fibrotic, metabolic, and behavioral abnormalities [57,58]. The findings suggest that merely restoring TM cellularity will not always bring conventional outflow back to normal, especially when the downstream endothelial component is dysfunctional. The finding that selected iPSC-derived regenerative cells can affect both TM and SC [40] is highly relevant. It indicates that the conventional outflow pathway is better conceptualized as a coupled TM–SC biological unit rather than as two serial and independent structures.

The extracellular mechanical environment is also important. A transplanted cell does not enter a normal TM with empty cellular niches; it enters tissue with abnormal ECM composition, increased stiffness, altered cytoskeletal tension, and dysregulated mechanotransduction. YAP/TAZ signaling in human trabecular meshwork (TM) cells has been shown to respond to substrate stiffness and TGF-β2 [59]. Additionally, SC endothelial cells respond in a comparable stiffness-dependent manner [60]. Normal trabecular meshwork (TM) cells and glaucomatous TM cells respond differently to the mechanical properties of the surrounding matrix [61]. These observations raise an important concern for cell replacement strategies: even a correctly differentiated cell may not maintain a normal phenotype when placed into a persistently pathological niche.

Conversely, the diseased niche seems to remain biologically modifiable. Experimental matrix softening can reverse certain aspects of abnormal SC mechanosignaling [60]. Pharmacological inhibition of ROCK with netarsudil can attenuate fibrosis, tissue stiffening, and outflow dysfunction in glucocorticoid-induced ocular hypertension [64]. There have been no combination experiments demonstrating that pharmacological treatment enhances stem-cell transplantation. Nevertheless, these findings lend credence to the idea of investigating regenerative strategies that consider both cellular therapy and microenvironmental remodeling. Bioengineered systems such as three-dimensional matrices, hydrogels, human anterior-segment organ culture, and other systems may be useful for assessing whether transplanted cells remain functionally stable under glaucomatous mechanical conditions before long-term in vivo experiments [62,63].

Delivery adds another layer to this interaction. Intracameral administration is a relatively simple way to access the outflow pathway, but it does not ensure cell localization, retention, or circumferential coverage by itself. The ability to steer labeled cells using magnetic approaches enhances their targeting to the TM [43,44]. Overall, the prolonged pressure response obtained with magnetically steered cells, which occurs despite limited donor-cell persistence, accords with the broader concept of host-mediated or paracrine regeneration [45]. In the same study, the tumorigenicity signal observed with the iPSC-derived preparation is a critical counterweight to the efficacy data [45]. This should not be generalized to all iPSC-derived TM products, because tumor risk depends on cell preparation, differentiation, purity, and manufacturing conditions. Nonetheless, it illustrates why increasing therapeutic potency cannot be considered independently of lineage stability and long-term safety.

The various cell sources therefore present distinct rather than interchangeable advantages. Tissue-specific TMSCs have intrinsic TM tropism and regenerative activity, although they are rare and their frequency diminishes with age [17,18]. Although MSCs are more accessible and seem particularly capable of paracrine modulation, their molecular and functional identity differs substantially from native TM, making differentiation and potency criteria essential [28,29,30,31,32]. iPSCs provide an expandable and potentially standardized source from which defined regenerative subpopulations can be generated, but pluripotent-cell-derived products require stringent control of residual undifferentiated cells and phenotypic stability [33,34,35,36,37,38,39,40,45]. The use of neural crest-derived populations provides a developmental rationale for TM specification, but the current evidence from in vivo studies is limited and short-term [41,42]. The available studies do not identify a single superior cell source; rather, they suggest that source, differentiation state, regenerative mechanism, delivery method, and safety profile must ultimately be considered as components of the same therapeutic product.

Accordingly, translational readiness should not be ranked solely according to the magnitude of experimental IOP lowering. Tissue availability and expansion capacity favor MSC- and pluripotent-cell platforms, whereas tissue specificity favors TMSCs; however, phenotype definition, potency assays, genomic stability, tumorigenicity, immunogenicity, and delivery reproducibility remain incompletely standardized across all platforms. No current cell product can therefore be considered clinically mature.

These considerations become particularly important when moving from experimental efficacy to clinical-grade manufacturing. Ocular regenerative medicine experience shows that defining cell identity, purity, potency, stability, and long-term behavior is critical before any transplantation product can be evaluated clinically [65]. For chronic diseases with a low tolerance for therapeutic risk, factors including reproducibility across donors and production batches, differentiation control, genetic stability, and the effects of expansion and storage become particularly significant. More broadly, clinical experience with stem-cell therapies has shown the potential consequences of inadequately controlled interventions, which underscores the value of rigorous manufacturing, regulatory oversight, and long-term safety surveillance. The evaluation of immunogenicity will also need to be cell-source-specific. Further, engineering of cells to escape immune recognition can itself affect safety and tumor surveillance. This reinforces the need to assess efficacy and biological containment together [66].

Safety considerations also differ across cell platforms. For TMSCs, MSCs, and other adult-cell products, relevant concerns include ectopic localization to the corneal endothelium, iris, lens, or non-target angle structures; inflammatory responses; fibrosis; angle obstruction; and uncontrolled or persistent proliferation. Pluripotent-cell-derived products additionally require rigorous exclusion of residual undifferentiated cells, genomic instability, and tumorigenic potential. Magnetically targeted products introduce further questions regarding nanoparticle persistence, intracellular processing, and long-term tissue exposure. These issues are particularly important in the anterior chamber, where treatment may be difficult to retrieve or reverse after administration. Repeated dosing would also require assessment of cumulative inflammatory, immunological, and biodistribution effects rather than assuming that the safety profile of a single administration remains applicable.

Clinical translation requires an endpoint framework that distinguishes regeneration from traditional IOP lowering. A decrease in IOP represents necessary evidence of functional benefit; however, it is not in itself evidence that regeneration of an outflow pathway has occurred. IOP can already be reduced through surgery, laser therapy, and drugs without reconstructing the native TM–SC system. A first-in-human regenerative program should therefore include, at minimum, assessment of safety, IOP, medication burden, conventional outflow function, durability of effect, structural evaluation of the angle and TM–SC complex where technically feasible, and evidence of biological target restoration. These outcomes should be interpreted in two separate domains: functional efficacy, demonstrated by sustained improvement in pressure control and outflow function, and proof of regeneration, demonstrated by restoration of the intended cellular, structural, or molecular target. Tonographic and OCT-based approaches have been proposed for evaluating outflow-tissue function after regenerative intervention [66]. Reduced dependence on additional pressure-lowering therapy and durability after a single intervention would also be clinically relevant. However, these outcomes are not substitutes for the demonstration of biological target restoration.

The term “beyond pressure lowering” should therefore be carefully interpreted. The existing literature does not demonstrate any clinically significant IOP-independent neuroprotection related to TM or SC regeneration. After the successful restoration of outflow in experimental glaucoma models, retinal ganglion cell preservation is compatible with protection secondary to improved IOP control [24,35]. However, these observations cannot, on their own, indicate a direct neuroprotective action of the transplanted cells. In order to establish such an effect, experimental designs would be required in which pressure-dependent and pressure-independent mechanisms are explicitly separated. The term “beyond pressure lowering” essentially refers to biological, rather than neuroprotective, effects. These include restoration of cellularity, endogenous regenerative capacity, extracellular homeostasis, tissue biomechanics, and TM–SC communication rather than chronic manipulation or bypass of aqueous humor resistance.

The next stage of the field should be guided by several limitations of the available evidence. Many studies remain preclinical, while they differ markedly in species, mechanism of glaucoma, degree of TM injury, cell source, differentiation protocol, dose administered, delivery method, and duration of follow-up. Different biological questions are addressed by laser-induced damage, transgenic myocilin glaucoma, pharmacological ocular hypertension, experimentally induced cell loss, and normal-eye transplantation, and these models should not be treated as interchangeable with human POAG. Perfusion models of the human anterior segment are an important link but cannot replicate systemic immunity, vascular biology, or long-term host responses.

Therefore, future research must move beyond demonstrating only the possibility of lowering IOP with a certain cell population. Candidate cell products should be tested comparatively under standardized conditions and have lineage tracing included to distinguish between donor-cell replacement and host–cell proliferation. By using single-cell and spatial approaches, it may be possible to identify which endogenous TM and SC populations are restored and whether regeneration reproduces normal cellular states rather than merely increasing cell number. Long-term experiments should establish biodistribution, persistence, immunogenicity, genomic stability, and tumorigenicity. Dose–response experiments should determine the minimum cell exposure needed for durable repair. Ultimately, a direct comparison of viable-cell therapy with secretome- or extracellular-vesicle-based approaches, along with experimental combination with controlled ECM or mechanobiological modulation, will be important to determine whether durable restoration of aqueous outflow depends on transplantation, endogenous repair, glaucomatous niche remodeling, or a combination of these processes.

The existing evidence suggests that regeneration of the conventional aqueous outflow pathway is a biologically plausible, although still preclinical, strategy. The possibility of simply replacing missing TM cells is no longer its most compelling feature. The new, emerging findings instead point to a regenerative system by which appropriately chosen donor cells can interact with residual host cells, the extracellular matrix, and Schlemm’s canal to re-establish components of physiological outflow regulation. The main challenge is determining whether these experimental effects can be translated into a defined, reproducible, and safe therapeutic product that restores durable outflow homeostasis in human glaucoma.

## 5. Conclusions

Regeneration of the conventional aqueous outflow pathway is emerging as a biologically distinct approach to glaucoma treatment. Preclinical evidence suggests that regenerative effects may extend beyond direct replacement of lost TM cells. A combination of donor-cell integration, stimulation of endogenous TM-cell proliferation, paracrine signaling, extracellular-vesicle-mediated effects, and remodeling of the extracellular and mechanical microenvironment may contribute to regenerative effects. The restoration of TM cellularity alone may not suffice if Schlemm’s canal dysfunction and the abnormal glaucomatous extracellular matrix are not simultaneously addressed. The evidence boundary should, however, remain clear. Durable reconstruction of the human glaucomatous TM–SC unit has not been demonstrated, the optimal therapeutic cell identity remains undefined, long-term safety has not been established, and no regenerative strategy has yet shown superiority over existing outflow-directed treatments. Clinical translation therefore requires more than manufacturing optimization: it requires reproducible evidence that a well-defined cell product can safely restore biological target tissue and durable outflow function in the human eye.

## Figures and Tables

**Table 1:** Stem and progenitor cell-based strategies for regeneration of the conventional aqueous outflow pathway.

Cell Source/Strategy	Evidence Level/Experimental Model	Main Regenerative Findings	Functional Outflow Evidence/Follow-Up	Main Limitations/Unresolved Issues	References
Trabecular meshwork stem/progenitor cells (TMSCs)	In vitro: isolated/expanded human TM-derived populations. In vivo normal eye: intracameral transplantation in mice. In vivo injury/disease models: laser-damaged TM and Tg-MYOC^Y437H glaucoma model. Ex vivo human: perfused anterior-segment cell-loss model.	Human TM-derived stem/progenitor populations show clonogenic/multipotent properties and can acquire TM-associated markers and phagocytic function. After intracameral delivery, transplanted cells preferentially localize to the TM; α5β1 contributes to retention, whereas CXCR4/SDF1 contributes to injury-directed homing. In glaucoma and cell-loss models, transplantation can increase TM cellularity and stimulate endogenous TM-cell proliferation.	Functional restoration demonstrated in disease/cell-loss models. In Tg-MYOC^Y437H mice, transplantation reduced IOP and increased conventional outflow facility with structural TM improvement. In human organ-cultured anterior segments, TMSC transplantation increased outflow facility after experimentally induced TM-cell loss. Persistence of transplanted cells has been demonstrated for up to several months in normal mouse eyes; functional follow-up varies by model.	TMSC identity is not standardized and reported populations are partially overlapping. Autologous availability may be limited by age-associated depletion. Long-term persistence, potency criteria, manufacturing standardization, and efficacy in human glaucoma remain unresolved.	Identity/differentiation: [15,16]. Normal-eye localization: [17]. Resident population/heterogeneity: [18,19,20,21]. Homing/retention: [22,23]. Disease-model functional rescue: [24]. Human ex vivo rescue: [25]. Paracrine mechanisms: [26,27].
Mesenchymal stem/stromal cells (MSCs)	In vivo disease model: laser-induced ocular hypertension. In vitro: cultured human TM cells exposed to MSC-conditioned medium or MSC-derived extracellular-vesicle preparations.	MSCs can promote tissue repair through secreted factors and stimulation or recruitment of endogenous progenitor-like cells. Conditioned medium from appropriately stimulated MSCs can reproduce part of the biological response. MSC-derived vesicular preparations reduce oxidative and inflammatory injury and modify TM-cell survival, phagocytosis, and matrix-related signaling.	Functional evidence present but platform-dependent. IOP lowering and tissue repair have been reported in experimental ocular-hypertension models, including effects reproduced partly by conditioned medium. Vesicle studies are predominantly mechanistic/in vitro and do not establish restoration of conventional outflow in vivo.	MSCs are not molecularly or functionally equivalent to native TM cells. Potency assays, differentiation state, secretome composition, biodistribution, dose, duration of action, and long-term safety remain incompletely defined.	Cell/conditioned-medium efficacy: [28]. MSC–TM phenotypic comparison: [29]. Extracellular-vesicle studies: [31,32].
Adipose-derived stem cells/TM-directed ADSC derivatives	In vivo normal eye: intracameral transplantation of undifferentiated ADSCs and ADSC-TM cells. In vivo disease model: magnetically targeted cell delivery in experimental glaucoma/outflow dysfunction.	Both undifferentiated ADSCs and TM-directed derivatives can localize to the TM. TM-directed differentiation generates TM-associated transcriptional, phagocytic, and dexamethasone-responsive properties. Magnetic nanoparticle-based targeting substantially increases circumferential TM localization and permits delivery of relatively low cell numbers.	Normal-eye studies: compatibility/localization demonstrated without therapeutic rescue because baseline outflow was normal. Disease-model efficacy: magnetically targeted human adipose-derived MSCs produced sustained IOP reduction associated with increased conventional outflow and TM cellularity; reported effect persisted for 9 months.	Therapeutic mechanism may depend more on paracrine activity than durable donor-cell persistence. Optimal differentiation state, dose, nanoparticle safety, scalability, targeting reproducibility, and applicability to the human anterior chamber remain uncertain.	TM-directed differentiation/normal-eye localization: [30]. Magnetic targeting: [43,44]. Long-term functional efficacy: [45].
iPSC-derived TM-like cells	In vitro differentiation. Ex vivo human: experimentally depleted and perfused human anterior segments. In vivo disease models: Tg-MYOC^Y437H and aged glaucomatous mice.	iPSCs can be differentiated into TM-like cells showing TM-associated morphology, phagocytosis, dexamethasone responsiveness, and matrix-regulatory functions. Following transplantation, restoration of TM cellularity is driven substantially by proliferation of endogenous TM cells rather than extensive donor-cell replacement. Connexin-43-dependent donor–host communication contributes experimentally to this response.	Functional restoration demonstrated. Restoration of IOP homeostasis/outflow regulation has been shown in ex vivo and glaucoma models. In Tg-MYOC^Y437H mice, reduced IOP and increased outflow facility persisted for at least 9 weeks, with efficacy also demonstrated at 12 weeks in older animals. In perfused aged human donor eyes, increased TM cellularity occurred despite donor cells constituting <1% of the regenerated population.	Differentiation heterogeneity, residual pluripotency, genomic stability, tumorigenicity, product purity, lineage stability, and manufacturing reproducibility are major translational concerns.	Differentiation: [33]. Ex vivo functional restoration: [34]. Glaucoma-model rescue/endogenous proliferation: [35,36]. Human donor-eye repopulation: [37]. Connexin-43 mechanism: [38].
ITGA6-positive iPSC-derived regenerative population	Preclinical mechanistic studies: multi-omics characterization and experimental regenerative assays in outflow models.	ITGA6-positive iPSC-derived cells exhibit a transcriptional state distinct from mature primary TM cells and show enhanced capacity to stimulate endogenous TM-cell proliferation and repopulation of TM and SC. Regenerative activity is associated with NEAT1 expression and paraspeckle biology; experimental modulation of paraspeckle assembly further enhances regenerative effects.	Regenerative activity demonstrated experimentally, including endogenous repopulation; however, evidence remains preclinical and does not yet establish durable therapeutic efficacy in human glaucomatous outflow tissue.	Requires independent validation, standardized cell production, defined potency criteria, durability studies, and long-term genomic/tumorigenicity assessment before translational relevance can be established.	[40]
hESC-derived neural crest cells	In vivo normal eye/integration study: rabbit anterior chamber. Safety-oriented in vivo study: NOD/SCID mouse tumorigenicity assessment.	Short neural crest induction generates HNK-1-, p75-, SOX10-, and AP-2α-positive cells. Following intracameral injection, cells integrate into TM tissue and acquire TM-associated markers including Aqp1, Chi3l1, and Timp3.	Therapeutic outflow restoration not demonstrated. IOP and central corneal thickness remained within normal ranges during the initial 2 weeks; no major adverse effects were reported during 5 weeks of rabbit follow-up. No teratomas were observed in the reported NOD/SCID safety assessment.	Evidence demonstrates short-term integration and lineage adaptation rather than rescue of established glaucoma. Ethical, immunological, manufacturing, residual pluripotency, and long-term safety issues remain.	Developmental rationale: [39,41]. In vivo transplantation/safety: [42].

Abbreviations: ADSC, adipose-derived stem cell; hESC, human embryonic stem cell; IOP, intraocular pressure; iPSC, induced pluripotent stem cell; MSC, mesenchymal stem/stromal cell; NOD/SCID, non-obese diabetic/severe combined immunodeficient; SC, Schlemm’s canal; TM, trabecular meshwork; TMSC, trabecular meshwork stem cell.

**Table 2:** Biological mechanisms and tissue-level targets underlying regenerative restoration of aqueous outflow.

Regenerative Axis	Evidence Supporting the Mechanism	Biological Significance for Outflow Regeneration	Mechanistic Support	Current Limitation	References
Endogenous TM regenerative reserve	Putative human TMSCs with high ABCG2 expression and a high nucleus-to-cytoplasm ratio are concentrated near Schwalbe’s line. Their proportion declines markedly with age, in parallel with overall TM cellularity. Additional stem/progenitor-like populations have been identified using p75 expression, clonogenicity, label retention, sphere formation, and differentiation assays.	Supports the presence of an age-sensitive resident stem/progenitor-like compartment that may contribute to TM maintenance or be recruited therapeutically.	Observational/associative. Human tissue studies establish localization, phenotype, and age-associated depletion, but do not demonstrate that loss of this population causes TM degeneration or glaucoma.	The reported populations are partially overlapping and are not defined by a universally accepted set of stem-cell criteria. In POAG tissue, reduction in ABCG2-high/p75-positive cells has not been conclusively established as an independent pathogenic event.	[18,19,20,21]
Homing and matrix-dependent integration	α5β1 integrin promotes TMSC attachment to fibronectin and long-term retention within the TM. In damaged TM, CXCR4/SDF1 signaling contributes to preferential recruitment of transplanted cells to injured regions.	Indicates that regenerative efficacy depends not only on cell administration, but also on effective localization and retention within target outflow tissue.	Direct perturbation evidence. Integrin blockade and experimental manipulation of CXCR4/SDF1 signaling alter attachment, retention, or injury-directed migration.	Molecular determinants of homing and retention in the human glaucomatous eye and their variability with disease severity remain incompletely defined.	[22,23]
Endogenous TM-cell proliferation	TMSC and iPSC-TM transplantation increases proliferation of host TM cells. In perfused human donor eyes, transplanted iPSC-TM cells constituted <1% of the repopulated TM despite increased overall tissue cellularity.	Indicates that donor cells may act as regenerative triggers rather than simply replacing missing cells one-for-one.	Functional evidence; causality partly established. Host–cell proliferation is directly observed after transplantation, but the relative contribution of specific donor-derived signals varies among models.	Relative contributions of donor-cell replacement and endogenous proliferation remain model- and cell-product-dependent. Durable host repopulation in human glaucoma has not been demonstrated.	[25,35,36,37,38]
Direct donor–host intercellular signaling	Connexin-43-dependent gap-junction communication and intercellular calcium transfer contribute to iPSC-TM-induced endogenous TM-cell proliferation.	Provides a mechanistic link between a relatively small donor population and a larger endogenous regenerative response.	Direct perturbation evidence. Pharmacological or genetic inhibition of connexin-43-dependent communication reduces donor-induced host–cell proliferation.	Demonstrated experimentally in preclinical models; persistence and importance of this mechanism in human glaucomatous tissue remain unproven.	[38]
Paracrine trophic signaling	TMSC-conditioned secretome attenuates oxidative and fibrotic stress in differentiated TM cells. HGF contributes to this effect, although inhibition of HGF signaling does not abolish the entire response. MSC-conditioned medium can also reproduce part of the biological effect of viable cells.	Suggests that regenerative potency may depend partly on secreted factors rather than durable donor-cell survival or terminal differentiation.	Direct perturbation for HGF; functional association for the broader secretome. HGF signaling has been experimentally inhibited, whereas the complete composition and causal contribution of the remaining secreted factors are incompletely defined.	The active secretome is complex; HGF represents only one component. Composition, potency assays, dose, pharmacokinetics, and duration of action remain undefined.	[26,28]
Extracellular-vesicle-mediated regeneration	TMSC-derived small extracellular vesicles enhance TM-cell survival, proliferation, migration, and resistance to oxidative stress. MSC-derived vesicular preparations improve phagocytosis, reduce cellular injury, and modify oxidative-stress-related pathways including NRF2 signaling.	Raises the possibility of a cell-free regenerative strategy capable of modifying endogenous TM function without persistent donor-cell engraftment.	Predominantly functional in vitro evidence. Biological effects have been demonstrated after vesicle exposure, but their sufficiency for restoring conventional outflow in vivo has not been established.	Pharmacokinetics, dose, biodistribution, tissue penetration, duration of action, vesicle identity, and in vivo efficacy remain incompletely defined.	[27,31,32]
Developmentally informed cell specification	Neural crest-based differentiation generates TM-like populations with dexamethasone responsiveness and cross-linked actin-network formation. hESC-derived neural crest cells can integrate into TM tissue and acquire TM-associated markers in vivo.	Developmental lineage may provide a rational route for producing cells compatible with the embryological origin and functional phenotype of native TM.	Functional differentiation evidence; limited in vivo integration evidence. Acquisition of lineage-associated features is demonstrated, but superiority over alternative differentiation routes has not been causally established.	The optimal differentiation stage for transplantation remains unknown. A mature TM-like phenotype may not correspond to maximal regenerative potency, and long-term therapeutic rescue has not been demonstrated.	[39,41,42]
Regeneration-inducing iPSC cell states	ITGA6-positive iPSC-derived cells exhibit a transcriptional state distinct from mature primary TM cells but show enhanced capacity to stimulate endogenous TM-cell proliferation and repopulation of TM and SC. Regenerative activity is associated with increased NEAT1 expression and paraspeckle abundance, and experimental enhancement of paraspeckle assembly further increases regenerative effects.	Suggests that regenerative potency may depend on a signaling-competent cell state rather than maximal resemblance to terminally differentiated TM cells.	Direct experimental manipulation. Manipulation of paraspeckle-associated biology alters regenerative activity, providing stronger mechanistic support than simple transcriptional association alone.	Findings require independent validation, standardized production, defined potency criteria, and long-term safety assessment before translational relevance can be established.	[40]
Targeted cell delivery	Magnetic nanoparticle labeling improves circumferential localization of transplanted cells to the TM. Optimized magnetic geometry further increases targeting efficiency. Long-term magnetic delivery achieved sustained IOP lowering with a relatively low cell dose.	Efficient targeting may reduce the number of cells required, increase local therapeutic exposure, and potentially limit off-target distribution.	Direct interventional evidence. Magnetic steering experimentally alters cell localization and delivery efficiency; long-term efficacy has also been demonstrated in a disease model.	Scalability to the human anterior segment, nanoparticle persistence and safety, reproducibility of circumferential targeting, and long-term biodistribution require further study.	[43,44,45]
TM–SC cellular heterogeneity	Single-cell studies identify multiple TM, SC, vascular, immune, and stromal populations. SC shows a lymphatic-biased endothelial identity with several transcriptional states and predicted ligand–receptor interactions with neighboring TM populations.	Indicates that regeneration should restore coordinated cellular states rather than simply increase the number of generic “TM-like” cells.	Observational/transcriptomic. Cellular states and predicted interactions are defined molecularly, but their necessity or sufficiency for successful regeneration has not been experimentally established.	Molecular atlases define candidate therapeutic targets but do not yet establish which cell states or cell–cell interactions are required for functional restoration.	[46,47,48,49,50]
Schlemm’s canal plasticity and rejuvenation	PROX1, ANGPT/Tie2 signaling, macrophage-derived vitronectin–integrin αvβ3 signaling, and related developmental pathways regulate SC identity and structure. Experimental reactivation or manipulation of these pathways can promote structural recovery or regrowth of aged SC. Exogenous stem cells can also be experimentally directed toward an SC-like endothelial phenotype.	Positions SC as a biologically modifiable component of the TM–SC functional unit rather than merely a passive downstream recipient of TM repair.	Direct genetic/pharmacological manipulation for endogenous SC plasticity; differentiation evidence for exogenous SC-like cells. These forms of evidence should not be equated with direct stem-cell reconstruction of SC.	Direct stem-cell-mediated reconstruction of SC remains substantially less developed than TM regeneration. A bona fide adult SC stem/progenitor population has not been established.	[51,52,53,54,55,56,57,58]
ECM stiffness and YAP/TAZ mechanotransduction	Increased matrix stiffness and TGF-β2 alter YAP/TAZ activity in TM cells. Stiffness-dependent YAP/TAZ activation also occurs in SC endothelial cells and can be experimentally reversed. Normal and glaucomatous TM cells show different mechanical responses to substrate stiffness.	A pathological ECM may alter the phenotype and function of both transplanted and endogenous cells; successful regeneration may therefore require correction of the recipient niche as well as cell replacement.	Direct mechanistic perturbation. Matrix stiffness and YAP/TAZ signaling have been experimentally manipulated, and reversal of stiffness-induced abnormalities has been demonstrated.	Direct evidence that combining mechanomodulation with stem-cell transplantation improves regenerative outcomes is still lacking.	[59,60,61]
Pharmacological remodeling of the regenerative niche	In experimental glucocorticoid-induced ocular hypertension, netarsudil attenuates fibrosis, tissue stiffening, IOP elevation, and outflow dysfunction. Bioengineered matrices and three-dimensional systems provide complementary platforms for studying cell–matrix interactions.	Demonstrates that pathological outflow biomechanics can be therapeutically modified and supports investigation of combined cellular and microenvironmental approaches.	Direct interventional evidence for pharmacological niche modification; supportive model-system evidence for bioengineered platforms.	Combination with regenerative cell therapy remains a biological rationale rather than a demonstrated therapeutic strategy.	[62,63,64]

Abbreviations: ECM, extracellular matrix; hESC, human embryonic stem cell; HGF, hepatocyte growth factor; IOP, intraocular pressure; iPSC, induced pluripotent stem cell; MSC, mesenchymal stem/stromal cell; NRF2, nuclear factor erythroid 2-related factor 2; POAG, primary open-angle glaucoma; SC, Schlemm’s canal; TAZ, transcriptional coactivator with PDZ-binding motif; TGF-β2, transforming growth factor beta 2; TM, trabecular meshwork; TMSC, trabecular meshwork stem cell; YAP, Yes-associated protein.

## Data Availability

No new data were created or analyzed in this study. Data sharing is not applicable to this article.
